# Expanding the knowledge on the diversity of the cavernicolous Styloniscidae Vandel, 1952 (Oniscidea, Synocheta) from Brazil, with descriptions of two new species from the semiarid karst regions

**DOI:** 10.3897/zookeys.1101.79043

**Published:** 2022-05-18

**Authors:** Ivanklin Soares Campos-Filho, Jéssica S. Gallo, Jonas E. Gallão, Dayana F. Torres, Yesenia M. Carpio-Díaz, Carlos Mario López-Orozco, Ricardo Borja-Arrieta, Stefano Taiti, Maria Elina Bichuette

**Affiliations:** 1 Department of Biological Sciences, University of Cyprus, Lefkosia (Nicosia), Cyprus University of Cyprus Lefkosia Cyprus; 2 Laboratório de Estudos Subterrâneos, Universidade Federal de São Carlos, São Carlos, São Paulo, Brazil Universidade Federal de São Carlos São Carlos Brazil; 3 Grupo de Investigación en Biología Descriptiva y Aplicada, Programa de Biología, Universidad de Cartagena, Campus San Pablo, Cartagena de Indias, Colombia Universidad de Cartagena Cartagena das Indias Colombia; 4 Istituto di Ricerca sugli Ecosistemi Terrestri, Consiglio Nazionale delle Ricerche, Sesto Fiorentino, Florence, Italy Istituto di Ricerca sugli Ecosistemi Terrestri, Consiglio Nazionale delle Ricerche Florence Italy; 5 Museo di Storia Naturale, Sezione di Zoologia “La Specola”, Florence, Italy Museo di Storia Naturale Florence Italy; 6 Grupo Bambuí de Pesquisas Espeleológicas, Belo Horizonte, Minas Gerais, Brazil Grupo Bambuí de Pesquisas Espeleológicas Belo Horizonte Brazil

**Keywords:** Açungui geomorphological group, Bambuí geomorphological group, Casa Nova geomorphological group, *Cylindroniscus*, Neotropical, *Pectenoniscus*

## Abstract

Two new species of *Pectenoniscus* from two caves in karst areas of the Brazilian semiarid region are described. *Pectenoniscuspankaru* Campos-Filho, Torres & Bichuette, **sp. nov.** from Gruna do Govi cave, Serra do Ramalho karst area, state of Bahia, and *Pectenoniscusfervens* Campos-Filho, Taiti & Bichuette, **sp. nov.** from Toca Coroa do Frade cave, Barra Bonita karst area, state of Piauí. In addition, specimens of *Cylindroniscusflaviae* from Gruta da Tapagem (= Caverna do Diabo), Açungui karst area were also recorded. An updated diagnosis of *Pectenoniscus* and a distribution map of the species examined herein are given.

## Introduction

Terrestrial isopods (Oniscidea) comprise approximately 4,000 species and more than 500 genera distributed in 38 families (Sfenthourakis and Taiti 2015; Dimitriou et al. 2019; Campos-Filho and Taiti 2021). The Oniscidea are one of the most representative taxa in the Brazilian subterranean environments, due to their favourable habitat conditions with high humidity and many different substrates and micro-habitats (Fernandes et al. 2016, 2019). To date, more than 210 species are known from Brazil, of which 70 have been recorded from caves. Among them, 31 species are considered troglobites (obligatory and restricted cave-dwellers) and several are troglophiles (facultative cave-dwellers) or trogloxenes (epigean species with individuals using subterranean resources) (Trajano 2012; Trajano and Carvalho 2017; Campos-Filho et al. 2018, 2019, 2020; Cardoso et al. 2020a, b, 2021). However, both troglobitic and troglophile species are not assigned with certainty to these categories due the lack of sampling outside caves. Endogean species exhibiting classical troglomorphic characters, such as lack or reduction of body pigments and eyes, might also occur in the unconsolidated substrate outside caves (Campos-Filho et al. 2014).

The family Styloniscidae comprises 120 species distributed in 18 genera (WoRMS 2021). The family has a worldwide distribution, with species inhabiting many terrestrial environments, including caves (Schmalfuss 2003). Fifty-eight species distributed in 13 genera have been recorded from caves, i.e., *Bamaoniscus* Taiti & Montesanto, 2020 (1 sp.), *Chaimowiczia* Cardoso, Bastos-Pereira, Souza & Ferreira, 2021 (2 spp.), *Clavigeroniscus* Arcangeli, 1930 (2 spp.), *Cordioniscus* Gräve, 1914 (15 spp.), *Cylindroniscus* Arcangeli, 1929 (5 spp.), *Indoniscu*s Vandel, 1952 (1 sp.), *Iuiuniscus* Souza, Ferreira & Senna, 2015 (1 sp.), *Pectenoniscus* Andersson, 1960 (7 spp.), *Spelunconiscus* Campos-Filho, Araujo & Taiti, 2014 (1 sp.), *Styloniscus* Dana, 1853 (7 spp.), *Thailandoniscus* Dalens, 1989 (3 spp.), *Trogloniscus* Taiti & Xue, 2012 (5 spp.), and *Xangoniscus* Campos-Filho, Araujo & Taiti, 2014 (8 spp.) (for all recorded species see Dalens 1987; Mulaik 1960; Schultz 1970, 1995; Green 1971; Vandel 1973, 1977, 1981; Ferrara and Taiti 1979; Taiti et al. 1992; Taiti and Howarth 1997; Schmalfuss and Erhard 1998; Andreev and Bozarova 2000; Andreev 2002; Green et al. 2002; Taiti and Xue 2012; Campos-Filho et al. 2014; Souza et al. 2015; Bastos-Pereira et al., 2017; Fernandes et al. 2018; Cardoso et al. 2020a, b, 2021; Taiti and Montesanto 2020).

To date, in Brazil, 26 species of the family distributed in nine genera have been recorded from caves, i.e., *Chaimowiczia* (2 spp.), *Clavigeroniscus* (1 sp.), *Cordioniscus* (1 sp.), *Cylindroniscus* (2 spp), *Iuiuniscus* (1 sp.), *Pectenoniscus* (8 spp.), *Spelunconiscus* (1 sp.), *Styloniscus* (2 spp.), and *Xangoniscus* (8 spp.) (Campos-Filho et al. 2018, 2019; Cardoso et al. 2020a, b, 2021). It is worth mentioning that the Styloniscidae in Brazil hold the highest diversity of troglobitic isopods comprising 20 out of 31 species (Campos-Filho et al. 2018, 2019, 2020; Cardoso et al. 2020a, b, 2021).

Two new species of *Pectenoniscus* from caves of two distinct Brazilian karst regions are described here. The first species comes from Gruna do Govi, Serra do Ramalho karst region, inserted in the Bambuí geomorphological group, state of Bahia, and the second from Toca Coroa do Frade, Barra Bonita karst region, Casa Nova geomorphological group, state of Piauí. In addition, specimens of *Cylindroniscusflaviae* Campos-Filho, Araujo & Taiti, 2017 from Gruta da Tapagem (also known as Caverna do Diabo), Açungui geomorphological group, are recorded here. An updated diagnosis of the genus *Pectenoniscus* is given to include the species described by Cardoso et al. (2020b) and the two new species. Ecological and conservation remarks considering IUCN threat categories, are provided.

## Materials and methods

### Collections and taxonomy

Specimens were collected by hand with the aid of tweezers and brushes and stored in 70% and 100% ethanol. Information about the microhabitat (entrance, twilight or aphotic zones) and environmental variables (temperature and relative air humidity) of the caves was also recorded. Descriptions are based on morphological characters with the use of micro-preparations in Hoyer’s medium (Anderson 1954). For each new species, the diagnosis, type material, description, etymology and remarks are given. The *habitus* images were taken with the stereomicroscope model Motic SMZ-168 and the Celestron Microcapture Pro. The photographs were prepared with Adobe Photoshop CC Lite (v. 17.1.1). The appendages were illustrated with the aid of a camera lucida mounted on a CH2 Olympus microscope. The final illustrations were prepared using the software GIMP (v. 2.8) with the method proposed by Montesanto (2015, 2016). A map highlighting the caves where all species occur, as well the hydrological attributes and pressures of economic activities in the region, is presented. The distribution map was constructed with the QGIS software (v. 3.18.1) and the final edition with PowerPoint Microsoft 365 (v. 2108).

The material is deposited in the scientific collection of cave fauna of the Laboratório de Estudos Subterrâneos (**LES**), Universidade Federal de São Carlos, São Carlos, Brazil.

### Study areas

#### Parque Estadual Caverna do Diabo, Açungui geomorphological group

The Açungui geomorphological group comprises the metamorphic limestone and dolomite rocks of ~ 600 million years ago, extending from south of the São Paulo state to north of the Paraná state (Rubbioli et al. 2019). Due to the altitudinal range, this group has one of the largest concentrations of irregular limestone areas in the country, including very ornamented caves crossed by rivers (Rubbioli et al. 2019). The Açungui group is located in the Chacoan subregion, in all provinces of the Parana domain, i.e., Atlantic Forest, *Araucaria* Forest, and Paraná Forest (Morrone 2014). According to Köppen’s criteria, it shows a warm temperate climate, fully humid with warm summer (Kottek et al. 2006).

**Figure 1.**
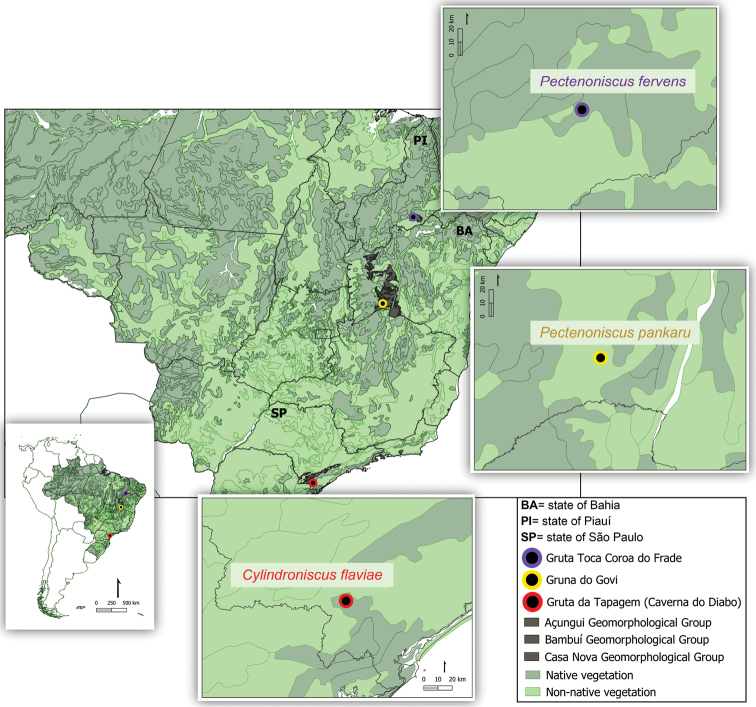
Distribution map of *Cylindroniscusflaviae* Campos-Filho, Araujo & Taiti, 2017, *Pectnenoscispankaru* Campos-Filho, Torres & Bichuette, sp. nov., *Pectenonscusfervens* Campos-Filho, Taiti & Bichuette, sp. nov.

**Figure 2.**
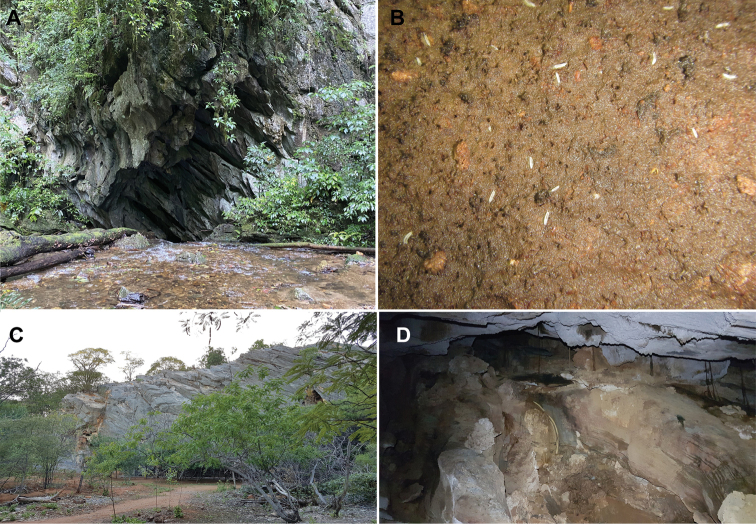
**A** Gruta da Tapagem (Caverna do Diabo), Açungui geomorphological group **B***Cylindroniscusflaviae* Campos-Filho, Araujo & Taiti, 2017 foraging in the organic matter **C** surrounding area outside Gruta Toca Coroa do Frade, Casa Nova geomorphological group **D** aphotic zone of the Toca Coroa do Frade.

This region includes the Caverna do Diabo State Park (PECD, in Portuguese, Parque Estadual da Caverna do Diabo), state of São Paulo (Fig. 1), which covers the municipalities of Barra do Turvo, Cajati, Eldorado and Iporanga (Fundação Florestal, 2010). The PECD was created in 2008 and it has more than 40,000 ha, constituting the Jacupiranga mosaic of conservation units (Fundação Florestal 2010). Gruta da Tapagem, also known as Caverna do Diabo (Fig. 2A), is ~ 8 km long and it is considered one of the most important caves of the PECD. The cave is inserted in the Tapagem dolomitic marble, in the André Lopes carbonate belt, and it is a sinkhole of the Ribeirão da Tapagem, a river which develops its subterranean course in ~ 4 km to the resurgence in the Vale do Rio das Ostras, a right-bank tributary of the Ribeira de Iguape (Karmann and Sánchez 1979; Hiruma et al. 2008; Rubbioli et al. 2019). The temperature and relative humidity of the air of the cave ranged from 28.8 °C and 60% RH in the entrance zone to 26.5 °C and 78% RH in the aphotic zone. This cave is notable for its scenic beauty with large halls and speleothems, and a stretch with illumination, stairs, and walkways for touristic activity (Silverio 2014).

#### Serra do Ramalho karst area, Bambuí geomorphological group

The Bambuí geomorphological group has the largest limestone area (ca. 146,000 km²) and the highest number of caves in Brazil (Rubbioli et al. 2019). The group includes the Serra do Ramalho karst area, located in the southwestern of the state of Bahia and the municipalities of Coribe, Feira da Mata, Carinhanha and Serra do Ramalho (Rubbioli et al. 2019). This area is inserted in the middle of the São Francisco River basin, dominated by a plateau of carbonate rocks with a high number of caves, mostly without legal protection (Auler et al. 2001; Rubbioli et al. 2019). According to Köppen’s criteria, the climate is tropical dry, characterised by dry winters and annual precipitation of ~ 640 mm (Bedek et al. 2018, 2020). The dominant vegetation is “Caatinga”, composed of mesophytic and xeromorphic forests interspersed with “Cerrado” (savannah-like vegetation) (Bichuette and Rizzato 2012).

**Figure 3.**
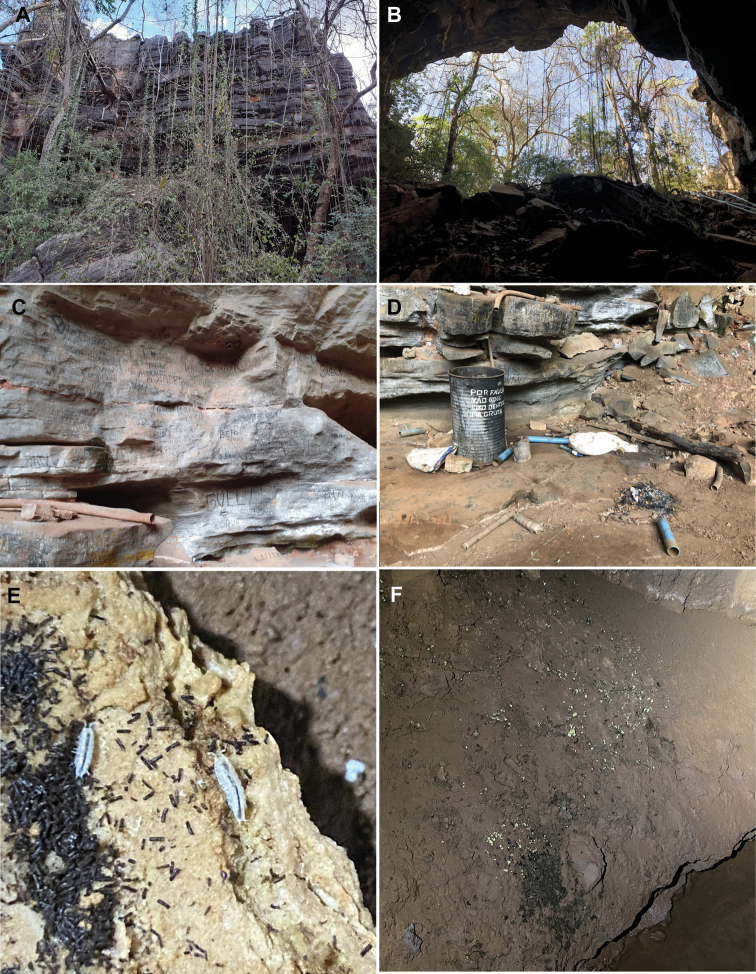
Gruna do Govi, Bambuí geomorphological group **A** surrounding area outside the cave **B** cave entrance **C, D** outside cave illustrating the anthropic impacts **E***Pectenoniscuspankaru* Campos-Filho, Torres & Bichuette, sp. nov. foraging in the organic matter **F** cave habitat where the specimens were collected.

The Gruna do Govi (Figs 1, 3) is located in a private property of the municipality of Feira da Mata. The surrounding of the cave harbours native vegetation and pastures, and anthropic impacts like garbage, graffiti on the walls and systems for capturing water from the subterranean drainage (Fig. 3C, D).

#### Barra Bonita karst region, Casa Nova geomorphological group, state of Piauí

The Barra Bonita karst region is inserted in the Casa Nova geomorphological group, and it is formed by quartzites, mica schists, and limestones of ~ 740 Myr (Rubbioli et al. 2019). The limestone area has a restricted occurrence in southeastern of the Piauí state, surrounded by “Caatinga” as typical vegetation domain, annual temperatures ranging from 25 °C to 31 °C, and 689 mm of average annual precipitation (Nascimento & Mantesso-Neto 2013; Hadler et al. 2018).

The Gruta Toca Coroa do Frade (Figs 1, 2C, D) is located in the municipality of Coronel José Dias, outside of the Parque Nacional da Serra da Capivara. The temperature and relative humidity of the air of the cave ranges from 30.9 °C and 49% RH in the entrance zone to 31 °C and 63% RH in the aphotic zone.

## Systematic account

### Genus Cylindroniscus

Taxon classificationAnimaliaIsopodaStyloniscidae 

Arcangeli, 1929

C13B320F-F5FC-50AF-9BF3-EF99DA62F1E1

#### Type species.

*Cylindroniscusseurati* Arcangeli, 1929 by monotypy (see Schmidt and Leistikow 2004).

### Cylindroniscus flaviae

Taxon classificationAnimaliaIsopodaStyloniscidae 

Campos-Filho, Araujo & Taiti, 2017

869D6548-8EA8-5F05-BADB-869CADB376EE

Figs 1 , 2B

Cylindroniscus flaviae Campos-Filho, Araujo & Taiti, in Campos-Filho et al. 2017a: 229, figs 1–5.
Cylindroniscus flaviae ; Campos-Filho et al. 2017b: 70; Campos-Filho et al. 2018: 4; Fernandes et al. 2018: 441; Silva et al. 2018: 56.

#### Material examined.

Brazil●1♀, Gruta da Tapagem (Caverna do Diabo), Eldorado, Parque Estadual Caverna do Diabo, Açungui geomorphological group, state of São Paulo, 24°38'17.00"S, 48°24'4.00"W, leg. ME Bichuette, T Zepon, JE Gallão, 24.III.2021, LES 27755●1♀, same locality and collectors as for preceding, 24.III.2021, LES 27756●1♂, 1♀, same locality and collectors as for preceding, 24.III.2021, LES 27757●1♀, same locality and collectors as for preceding, 24.III.2021, LES 27758●2♀♀, same locality and collectors as for preceding, 24.III.2021, LES 27759●1♂, same locality and collectors as for preceding, 25.III.2021, LES 27760.

#### Remarks.

*Cylindroniscusflaviae* shows preference for organic matter deposits and highly humid areas in the aphotic zone (Fig. 2B). The organic matter was observed in several conduits of the Gruta da Tapagem, always far from the touristic stretches, and it was composed of particulate vegetal debris or small tree branches. The environmental variables along the cave ranged from 19.9 °C to 20.4 °C and the relative air humidity from 95% to 99.1%. The individuals demonstrated sensitivity to the flash lights of the lanterns, always moving in opposite direction.

#### Distribution.

This species is recorded from several caves in the Açungui geomorphological group (see Campos-Filho et al. 2017).

### Genus Pectenoniscus

Taxon classificationAnimaliaIsopodaStyloniscidae 

Andersson, 1960

ED9272AA-2C00-5A10-A8A2-63C10058F5B8

#### Type species.

*Pectenoniscusangulatus* Andersson, 1960 by monotypy (see Schmidt and Leistikow 2004).

#### Diagnosis.

After Andersson (1960) and Campos-Filho et al. (2019). Animals of reduced size, ≤ 3.5 mm. Body unpigmented and eyes absent. Body slender with lateral sides almost parallel. Dorsal surface of cephalon and pereon bearing small transverse tubercles, conferring granulated appearance, pleon smooth or slightly tuberculate. Cephalon with 4–6 rows of tubercles, gradually reducing in number from posterior to distal portion, pereonite 1 with two or three rows of tubercles, pereonites 2–7 with two rows of tubercles. Dorsal scale-setae triangular. Cephalon with antennary lobes and suprantennal line. Pleonites 3–5 epimera not developed (only developed in *P.angulatus*). Telson triangular with lateral sides concave and rounded apex. Antennula of three articles, distal article with aesthetascs arranged in one longitudinal row. Antennal flagellum of 3–5 articles. Mandibles with strong molar process, left mandible with two penicils, right mandible with one penicil (sometimes one penicil on molar process). Maxillula inner endite with three penicils, proximal one longest; outer endite composed of eight or nine teeth plus slender stalks. Maxilla of two lobes covered with thick and fine setae, inner lobe wider. Maxilliped basis with lateral sides almost parallel, endite rectangular bearing one stout penicil. Uropod protopod subquadrangular, exopod longer than endopod, protopod and exopod sometimes bearing glandular pores, endopod inserted proximally. Male pleopod 1 endopod of two articles, distal article flagelliform. Male pleopod 2 endopod consisting of two articles, distal portion stout bearing complex apparatus.

#### Remarks.

The genus *Pectenoniscus* was created by Andersson (1960) to allocate the new species *P.angulatus* from Itá, Nova Teutônia, state of Santa Catarina, Brazil. The author defined the genus by having the cephalon of “*Trichoniscus*-type”, dorsal surface of the cephalon and pereon with rounded tubercles, pleonites epimera large, left mandible with two penicils near lacinia mobilis, right mandible with one penicil near lacinia mobilis plus one in the molar process, maxillula outer endite composed of nine teeth and two slender stalks, inner endite of three penicils and proximal one longer than distal ones, genital papilla pear-shaped with tubelike termination, male pleopod 1 of “*Styloniscus*-type”, male pleopod 2 endopod with distal portion broad and bearing a comb-like formation, and male pleopod 5 exopods with a dorsal lobe to fit the pleopod 2 endopod. Campos-Filho et al. (2019) described *P.liliae* Campos-Filho, Bichuette & Taiti, 2019 from Caverna Chico Pernambuco, Coribe, Serra do Ramalho karst area, state of Bahia, and added some characters in the diagnosis of the genus. Recently, Cardoso et al. (2020b) described six new species from karst areas of the states of Bahia and Minas Gerais, increasing the knowledge on the diversity of the genus, that now comprises eight species, i.e., *P.angulatus*, *P.carinhanhensis* Cardoso, Bastos-Pereira, Souza & Ferreira, 2020, *P.iuiuensis* Cardoso, Bastos-Pereira, Souza & Ferreira, 2020, *P.juveniliensis* Cardoso, Bastos-Pereira, Souza & Ferreira, 2020, *P.liliae*, *P.montalvaniensis* Cardoso, Bastos-Pereira, Souza & Ferreira, 2020, *P.morrensis* Cardoso, Bastos-Pereira, Souza & Ferreira, 2020, and *P.santanensis* Cardoso, Bastos-Pereira, Souza & Ferreira, 2020.

### Pectenoniscus pankaru

Taxon classificationAnimaliaIsopodaStyloniscidae 

Campos-Filho, Torres & Bichuette sp. nov.

6C0F6470-F9E7-5B5C-A459-1425DB239723

http://zoobank.org/416BE93E-CA7C-4D98-9D6C-FE1BB265D264

**Figure 4.**
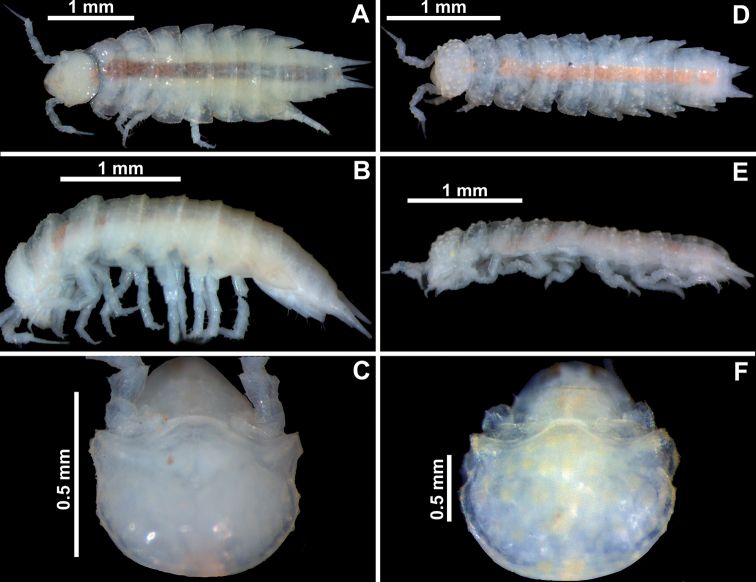
*Pectenoniscuspankaru* Campos-Filho, Torres & Bichuette, sp. nov. (♀, LES 27763) **A** habitus, dorsal view **B** habitus, lateral view **C** cephalon, dorsal view. *Pectenoniscusfervens* Campos-Filho, Taiti & Bichuette, sp. nov. (♀, LES 27764) **D** habitus, dorsal view **E** habitus, lateral view **F** cephalon, dorsal view.

**Figure 5.**
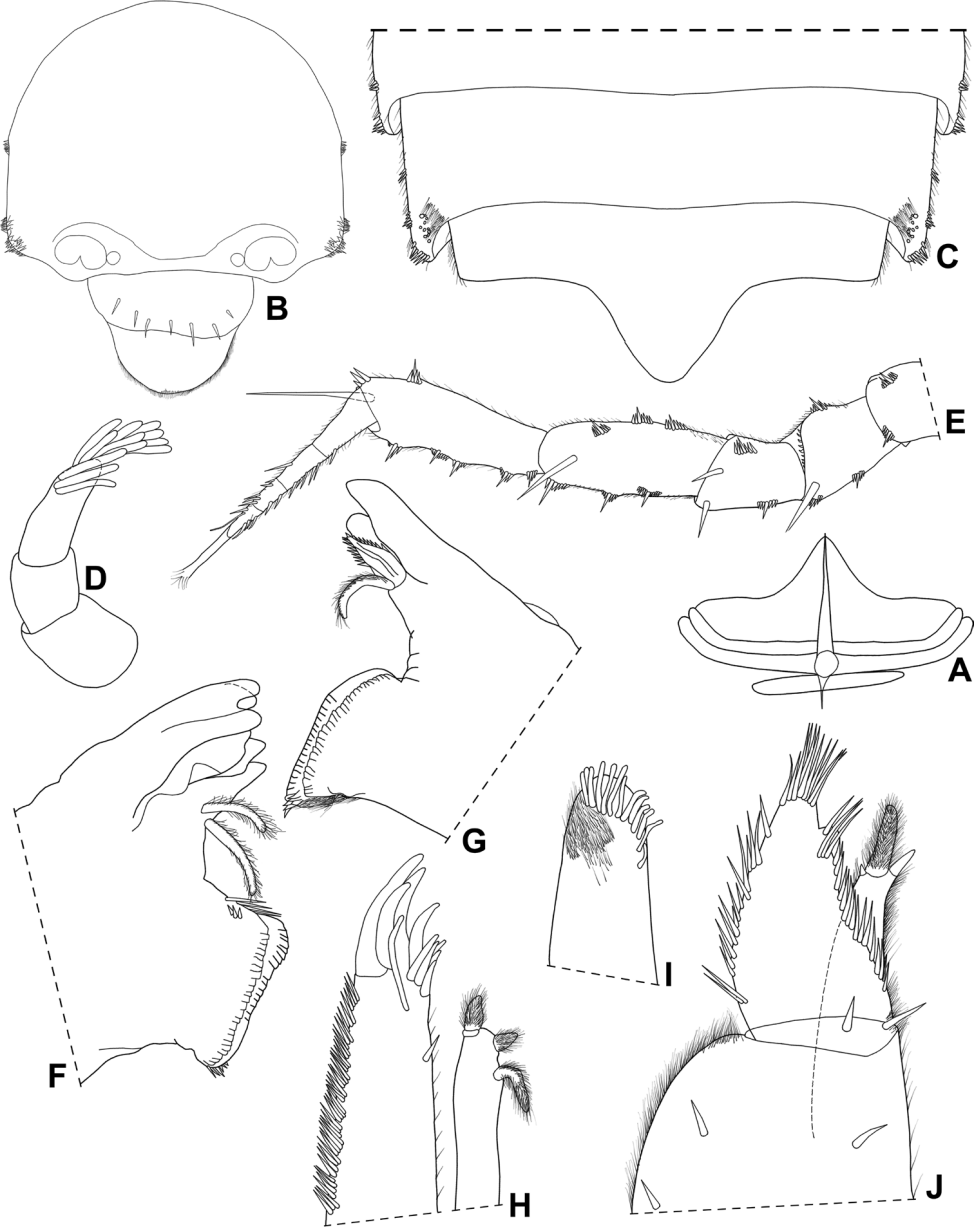
*Pectenoniscuspankaru* Campos-Filho, Torres & Bichuette, sp. nov. (♂, LES 27762) **A** dorsal scale-seta **B** cephalon, frontal view **C** pleonites 4, 5 and telson **D** antennula **E** antenna **F** left mandible **G** right mandible **H** maxillula **I** maxilla **J** maxilliped.

**Figure 6.**
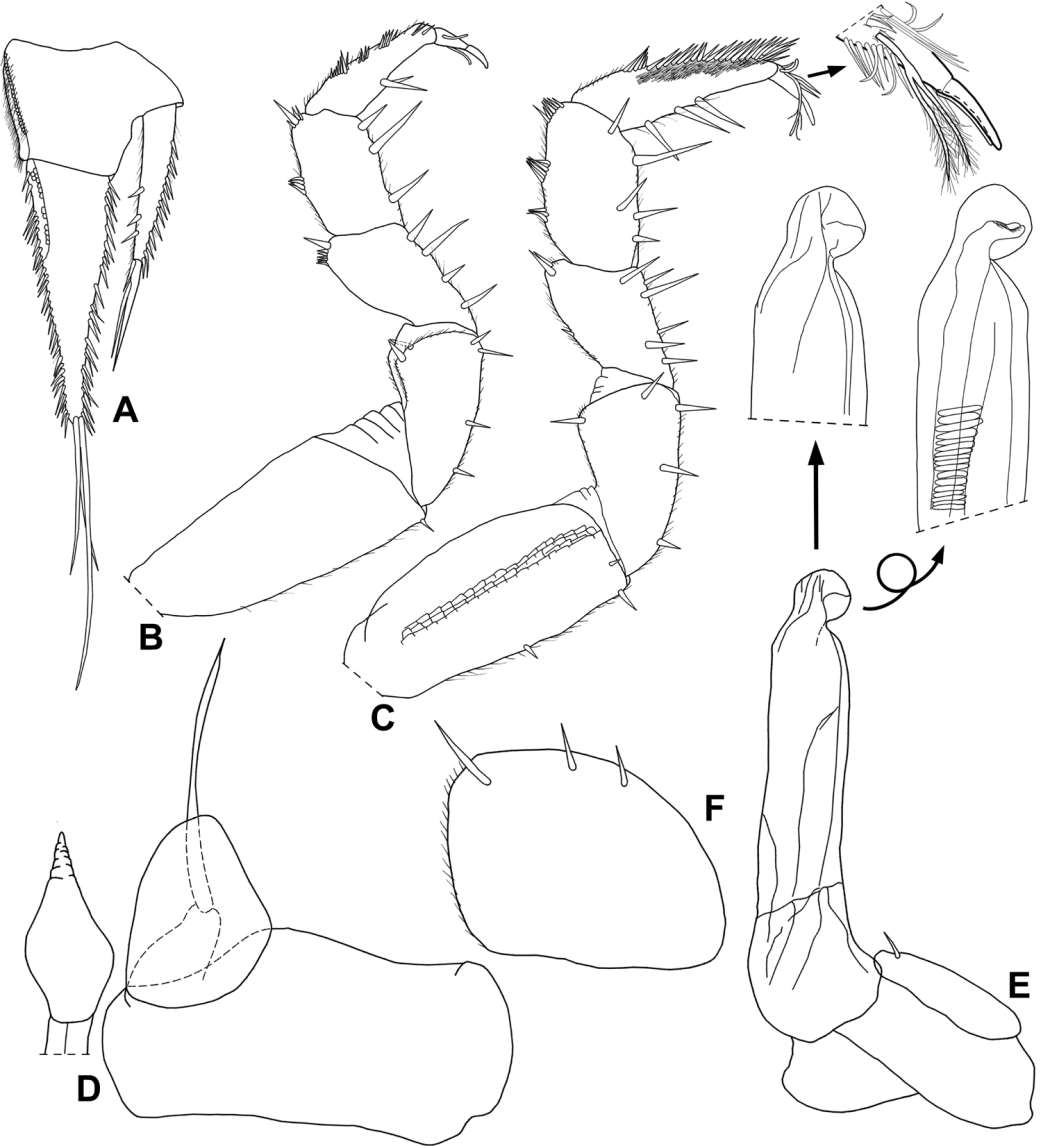
*Pectenoniscuspankaru* Campos-Filho, Torres & Bichuette, sp. nov. (♂, LES 27762) **A** uropod **B** pereopod 1 **C** pereopod 7 **D** genital papila and pleopod 1 **E** pleopod 2 **F** pleopod 5 exopod.

Figs 1 , 3E , 4A-C , 5 , 6

#### Material examined.

Brazil●1♂, ***holotype***, Gruna do Govi, Feira da Mata, Serra do Ramalho karst area, Bambuí geomorphological group, state of Bahia, 13°56'43.30"S, 44°14'25.94"W, 12.X.2020, leg. ME Bichuette, DF Torres, JS Gallo, LS Horta and JE Gallão, LES 27761●1♂ (parts in micropreparations), ***paratype***, same data as for holotype, LES 27762●2 ♀♀, ***paratypes***, same data as for holotype, LES 27763.

#### Description.

Maximum length: ♂ 2.2 mm, ♀ 3.5 mm. Dorsal surface slightly granulated, granules on pereonites 1–7 in two transverse rows, pleon smooth (Fig. 4A, B). Dorsal scale-setae tricorn-shaped (Fig. 5A). Cephalon (Figs 4C, 5B) with well-developed quadrangular antennary lobes, slightly directed outwards; profrons with suprantennal line bent downwards medially. Pereonite 1–3 epimera with postero-lateral corners right-angled, 4–7 progressively more acute and directed backwards; pleonite 5 epimera with glandular pores at sides near distal margins (Figs 4A, B, 5C). Telson (Fig. 5C) almost three times as wide as long, with concave sides and rounded apex. Antennula (Fig. 5D) with distal article longer than second and first, and bearing at least 12 aesthetascs. Antenna (Fig. 5E) with fifth article of peduncle as long as flagellum, bearing one distal strong seta; flagellum of four articles, first article longest, apical organ as long as distal article of flagellum. Mandibles as in Fig. 5F, G, right mandible with leaf-like lacinia mobilis. Maxillula (Fig. 5H) with two robust penicils; outer endite with 4+5 teeth, apically simple, one subapical slender stalk near medial margin. Maxilla as in Fig. 5I. Maxilliped (Fig. 5J) basis with lateral sides fringed with fine setae; palp with first article bearing two setae, distal articles fused and bearing many setae on lateral margins; endite much longer than wide, lateral margins covered with fine setae, distal margin bearing two strong setae and one elongated penicil. Grooves and scales for water conducting system on ischium, merus, carpus and propodus of pereopod 6 and basis of pereopod 7 (Fig. 6C). Dactylus with ungual seta simple and dactylar seta bifid and setose. Uropod (Fig. 6A) protopod and exopod grooved on outer margins bearing glandular pores; exopod longer than endopod, endopod inserted proximally.

**Male.** Pereopods 1–6 (Fig. 6B) without any sexual modifications. Pereopod 7 (Fig. 6C) propodus with brush of setae on rostral margin. Genital papilla (Fig. 6D) enlarged on median portion, apical part narrow and elongated. Pleopod 1 (Fig. 6D) protopod subrectangular, distal margin sinuous; exopod subtriangular, outer margin almost straight, proximal and outer margins convex; endopod longer than exopod, basal article short, distal article three times longer than basal one. Pleopod 2 (Fig. 6E) exopod ovoid, more than three times as wide as long; endopod of two articles, thickset, second article more than twice as long as first, distally bearing round shaped lobe directed outwards. Pleopod 5 exopod (Fig. 6F) subquadrangular, slightly wider than long, bearing three setae, distal margin rounded.

#### Etymology.

The new species is named for the indigenous people Pankaru, who inhabited the northern portion of Serra do Ramalho region.

#### Remarks.

*Pectenoniscuspankaru* sp. nov. differs from all other species of the genus in the shape of the male pleopod 2 endopod. Moreover, it differs in having the antennal flagellum composed of four articles (vs. five in *P.angulatus*, three in *P.carinhanhensis*, *P.iuiuensis*, *P.juveniliensis*, *P.lilae*, *P.montalvaniensis*, *P.morrensis* and *P.santanensis*), antennula bearing 12 aesthetascs (vs. six in *P.morrensis* and *P.santanensis*, eight in *P.angulatus* and *P.montalvaniensis*, nine in *P.juveniliensis* and *P.lilae*, ten in *P.iuiuensis*, and 11 in *P.carinhanhensis*) (see also Andersson 1960; Campos-Filho et al. 2019; Cardoso et al. 2020b).

Specimens of *Pectenoniscuspankaru* sp. nov. were found only in the aphotic zone associated to the sediment banks close to a small subterranean stream (Fig. 2B) and characterised by high humidity levels, which are more suitable for terrestrial isopods. The specimens were collected near vegetable debris.

### Pectenoniscus fervens

Taxon classificationAnimaliaIsopodaStyloniscidae 

Campos-Filho, Taiti & Bichuette sp. nov.

590F4ABE-A9D9-5C27-8BBC-75F8392A5C9E

http://zoobank.org/59D8BAB6-221D-47ED-8CE0-519D8A16E5DE

**Figure 7.**
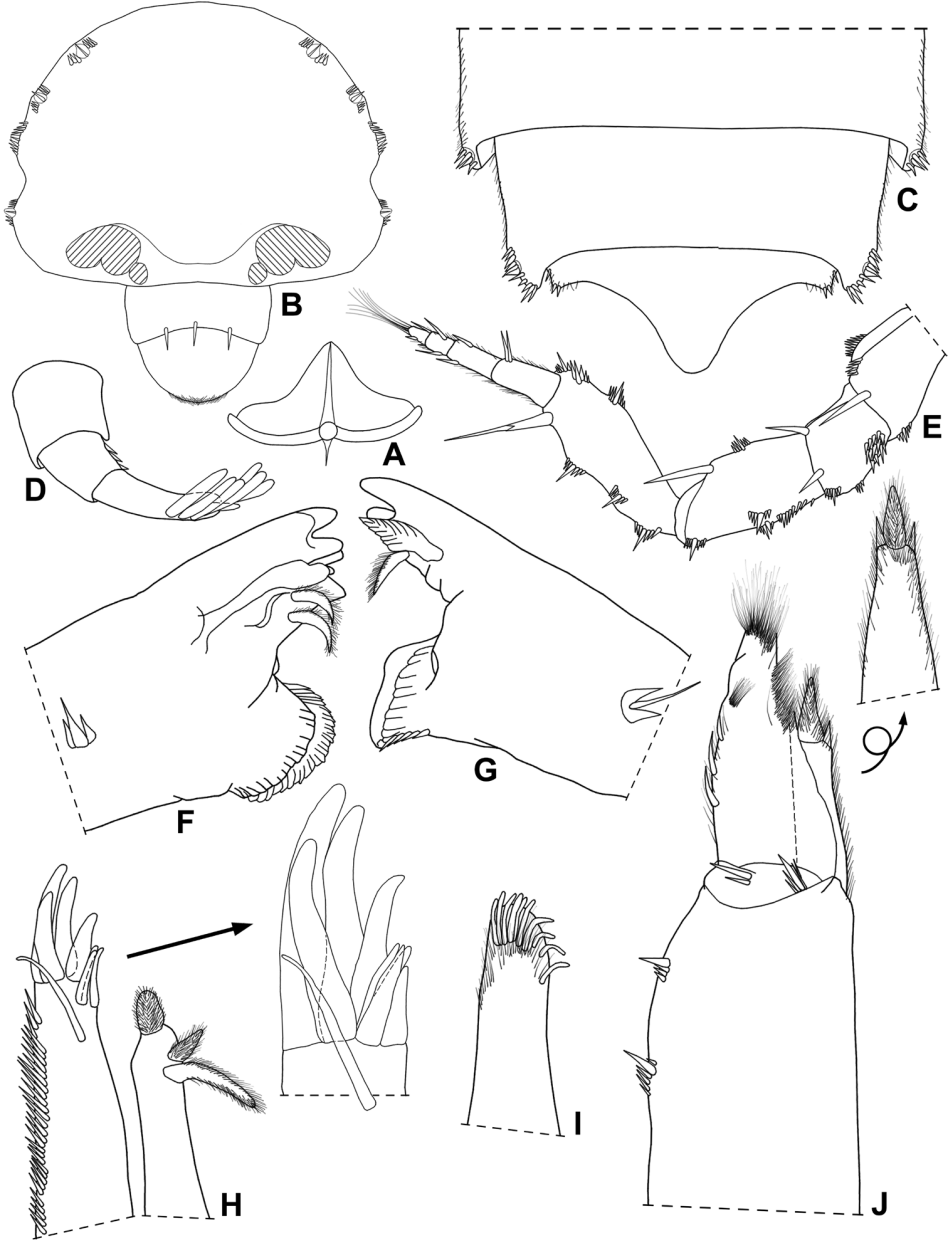
*Pectenoniscusfervens* Campos-Filho, Taiti & Bichuette, sp. nov. (♀, LES 27764) **A** dorsal scale-seta **B** cephalon, frontal view **C** pleonites 4, 5 and telson **D** antennula **E** antenna **F** left mandible **G** right mandible **H** maxillula **I** maxilla **J** maxilliped.

**Figure 8.**
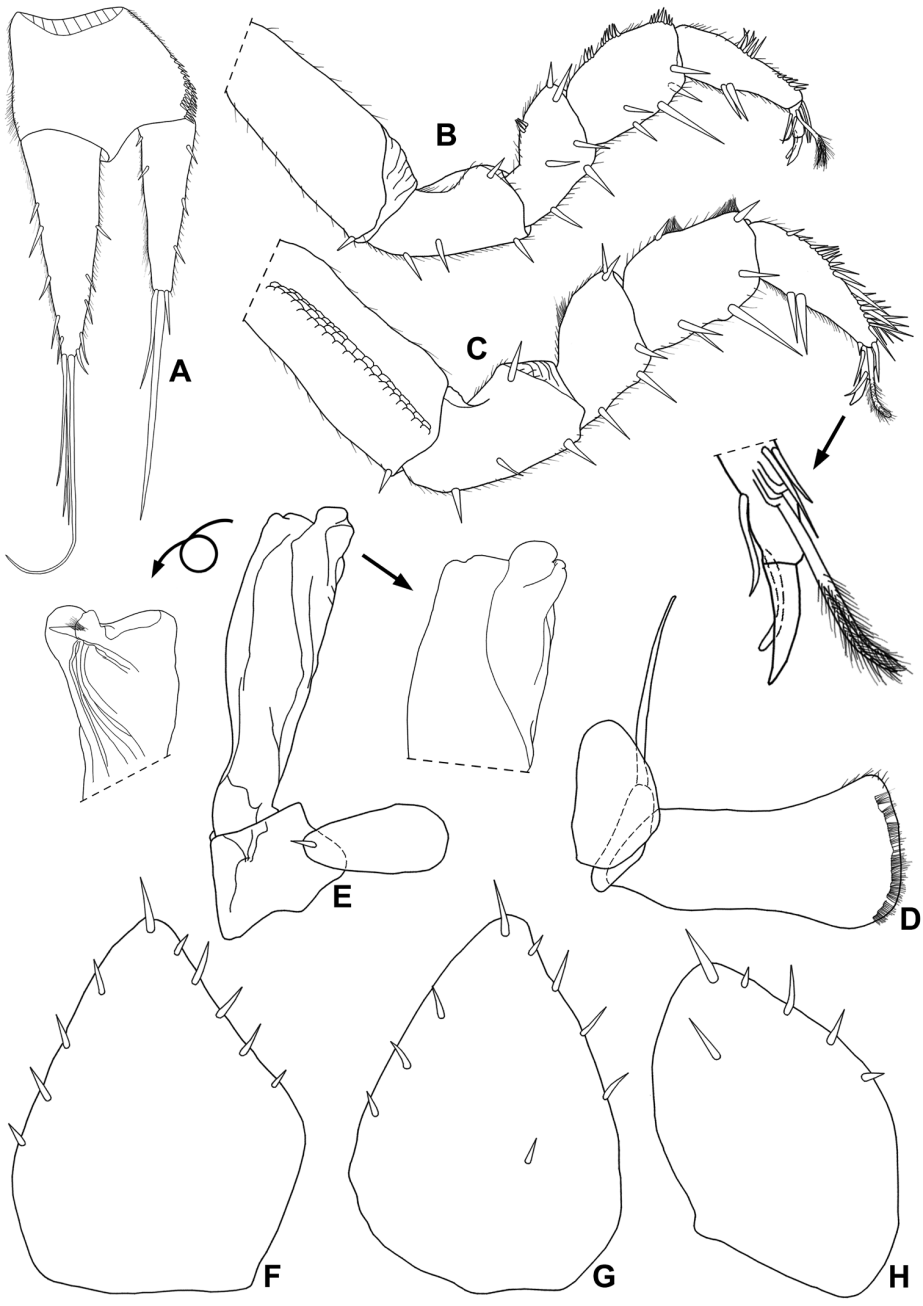
*Pectenoniscusfervens* Campos-Filho, Taiti & Bichuette, sp. nov. (♀, LES 27764) **A** uropod (♂, LES 22421) **B** pereopod 1 **C** pereopod 7 **D** pleopod 1 **E** pleopod 2 **F** pleopod 3 exopod **G** pleopod 4 exopod **H** pleopod 5 exopod.

Figs 1 , 2C, D , 4D, E , 7 , 8

#### Material examined.

Brazil●1♂ (parts in micropreparations), ***holotype***, Gruta Toca Coroa do Frade, Coronel José Dias, Barra Bonita karst region, Casa Nova geomorphological group, state of Piauí, 8°47'51.58"S, 42°25'1.47"W, 8.I.2018, leg. DM Schimonsky, DF Torres and JE Gallão, LES 22421●6♀♀ (one with parts in micropreparations), ***paratypes***, same data as for holotype, LES 27764.

#### Description.

Maximum length: ♂ and ♀ 3 mm. Dorsal surface granulated, granules on pereonites 1–7 in two transverse rows, on pleonites 3–5 in one row (Fig. 4D, E). Dorsal scale-setae tricorn-shaped in middle segments (Fig. 7A). Cephalon (Figs 4F, 7B) with antennary lobes small, triangular and slightly directed outwards; profrons with suprantennal line bent downwards medially. Pereonites 1 and 2 epimera with postero-lateral corners rounded, 4–7 progressively directed backwards and more acute (Fig. 4D, E). Pleonites 3–5 epimera without glandular pores (Fig. 7C). Telson (Fig. 7C) twice as wide as long, with concave sides and rounded apex. Antennula (Fig. 7D) with distal article longer than second and first, and bearing at least six aesthetascs plus distal tip. Antenna (Fig. 7E) with fifth article of peduncle slightly longer than flagellum, bearing one distal strong seta; flagellum of four articles, first and second articles subequal in length, third and fourth articles shorter; apical organ longer than distal article of flagellum. Mandibles as in Fig. 7F, G; right mandible with leaf-like lacinia mobilis. Maxillula (Fig. 7H) inner endite with apical penicil robust; outer endite of 4+5 teeth, apically simple, one subapical slender stalk near medial margin. Maxilla as in Fig. 7I. Maxilliped (Fig. 7J) basis with lateral sides fringed with fine setae; palp with first article bearing two setae, distal articles fused and bearing distal fringe of fine setae; endite much longer than wide, lateral margins covered with fine setae, distal margin bearing two strong setae and one elongated penicil. Grooves and scales for water conducting system on ischium, merus, carpus and propodus of pereopod 6 and basis of pereopod 7 (Fig. 8B). Dactylus with ungual seta simple and dactylar seta simple and apically setose. Uropod (Fig. 8A) protopod and exopod not grooved on sternal margin; exopod longer than endopod and inserted almost at same level.

**Male.** Pereopods 1–7 (Fig. 8B, C) without any sexual modifications. Genital papilla as in previous species. Pleopod 1 (Fig. 8D) protopod subrectangular, distal margin concave; exopod ovoidal, outer margin almost straight, proximal, inner and distal margins rounded; endopod longer than exopod, basal article short, distal article ca. twice longer than basal one. Pleopod 2 (Fig. 8E) exopod ovoidal, twice as wide as long, one seta on inner margin; endopod of two articles, thickset, second article more than three times longer than first, distal portion subquadrangular, distal outer margin with round shaped lobe directed outwards bearing one triangular process on ventral margin. Pleopod 3–5 exopods as in Fig. 8F–H.

#### Etymology.

Latin. *fervens* for very hot, boiling hot. The new species name refers to the very hot temperatures of the Brazilian state of Piauí.

#### Remarks.

*Pectenoniscusfervens* sp. nov. differs from all other species of the genus in the shape of the male pleopod 2 endopod. Moreover, it differs from *P.pankaru* sp. nov. in having the antennula bearing six aesthetascs, the dactylar seta stout and in the different shape of the male pleopod 1 and pleopod 3–5 exopods. The new species shows the same number of aesthetascs of the antennula as in *P.morrensis* and *P.santanensis*, from which it differs in the antennal flagellum composed of four articles (three in *P.morrensis* and *P.santanensis*), male pleopod 1 protopod with the distal margin concave (vs. straight in *P.morrensis*, almost straight in *P.santanensis*), male pleopod 4 exopod triangular (vs. subrectangular in *P.morrensis*, subquadrangualar in *P.santanensis*), and male pleopod 5 exopod rhomboid and longer than wide (vs. triangular and as long as wide in *P.morrensis*, subquadrangular in *P.santanensis*).

The specimens of *Pectenoniscusfervens* sp. nov. were found only in the aphotic zone (Fig. 2J), in vegetable debris with clay and under rocks, where the humidity was higher than at the cave entrance.

## Discussion

The new species of *Pectenoniscus* described here showed strict dependence on high humidity. Their occurrence in the caves present in semiarid regions, where the external temperatures are high and the humidity is low, reinforce the idea that these caves are probably important refuges for these animals due to their favourable conditions. Moreover, both species show remarkable troglomorphism, such as absent body pigments and eyes, which reinforces the classification of both species as troglobites.

A preliminary evaluation of the conservation status of the new species described here was carried out following the IUCN (International Union of Conservation of Nature) classification. *Pectenoniscuspankaru* sp. nov. and *Pectenoniscusfervens* sp. nov. were classified as Critically Endangered (CR) by the criteria B2ab(iii). The surrounding areas of the caves (Gruna do Govi and Gruta Toca Coroa do Frade) are impacted by deforestation and the remaining native vegetation is present only close to their entrances. The Serra do Ramalho karts area is historically threatened by agricultural activities and potential mining projects (Gallão and Bichuette 2018). Furthermore, the Gruna do Govi is used to capture subterranean water for the consumption of the local people and has pastures close to the cave, while the Gruta Toca Coroa do Frade, despite being close to the Parque Nacional da Serra da Capivara, is out of its boundaries and it is threatened by mining activities and increasing urbanisation. Moreover, both caves are not protected by any law.

## Supplementary Material

XML Treatment for Cylindroniscus

XML Treatment for Cylindroniscus flaviae

XML Treatment for Pectenoniscus

XML Treatment for Pectenoniscus pankaru

XML Treatment for Pectenoniscus fervens
